# Geometric Predictors of Knotted and Linked Arcs

**DOI:** 10.1021/acspolymersau.2c00021

**Published:** 2022-07-08

**Authors:** Joseph L. Sleiman, Robin H. Burton, Michele Caraglio, Yair Augusto Gutierrez Fosado, Davide Michieletto

**Affiliations:** † School of Physics and Astronomy, University of Edinburgh, Peter Guthrie Tait Road, Edinburgh EH9 3FD, United Kingdom; ‡ Institut für Theoretische Physik, Universität Innsbruck, Technikerstraße 21A, A-6020 Innsbruck, Austria; ¶ MRC Human Genetics Unit, Institute of Genetics and Cancer, University of Edinburgh, Edinburgh EH4 2XU, United Kingdom

**Keywords:** Polymers, Entanglements, Topology, Knots, Links, Writhe

## Abstract

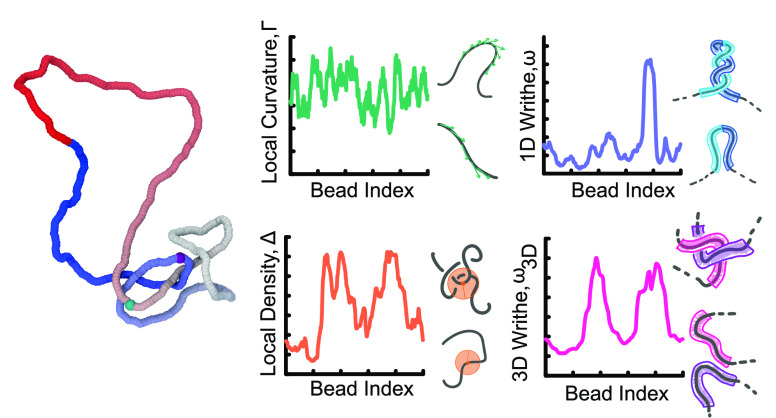

Inspired by how certain proteins “sense” knots and entanglements in DNA molecules, here, we ask if local geometric features that may be used as a readout of the underlying topology of generic polymers exist. We perform molecular simulations of knotted and linked semiflexible polymers and study four geometric measures to predict topological entanglements: local curvature, local density, local 1D writhe, and nonlocal 3D writhe. We discover that local curvature is a poor predictor of entanglements. In contrast, segments with maximum local density or writhe correlate as much as 90% of the time with the shortest knotted and linked arcs. We find that this accuracy is preserved across different knot types and also under significant spherical confinement, which is known to delocalize essential crossings in knotted polymers. We further discover that nonlocal 3D writhe is the best geometric readout of the knot location. Finally, we discuss how these geometric features may be used to computationally analyze entanglements in generic polymer melts and gels.

## Introduction

Topological entanglements are ubiquitous and an essential feature of everyday materials and complex fluids, endowing them with viscous and elastic properties. Entanglements are often poorly defined, and their unambiguous identification and quantification remain elusive.^1,2^ For example, a knot is a well-defined mathematical entity when tied on a closed curve, but there are many examples in physics and biology, e.g., proteins and chromatin, where knots are tied on open curves, rendering such “physical” knots much more difficult to define rigorously and unambiguously.^3−7^ More broadly, a long-standing goal in polymer physics and the broader soft matter communities is to understand and control the topology of certain systems from the geometry of (often entangled) 1D curves. This goal encompasses many fields, such as liquid crystals,^8^ optics,^9,10^ fluids,^11^ DNA,^6,12−18^ proteins,^19,20^ polymers,^3,21−23^ soap films,^24,25^ and soft matter in general.^26,27^ At the same time, the unambiguous characterization of entanglements in these systems is often elusive, in turn begging for better strategies to quantify entanglements in generic soft matter systems.

A striking example of the inherent difficulty in defining entanglements is seen in polymer melts, whereby the close contact of two chains does not necessarily indicate that chains are constraining each other’s motion. Instead, so-called “primitive”^28^ and “isoconfigurational”^29^ mean path techniques are far better placed to separate relevant entanglements from irrelevant ones. Yet, even these sophisticated techniques often struggle when polymers display nontrivial topology, e.g., rings.^30−32^ Ring polymers are in fact not amenable for standard primitive path analysis as they do not entangle in the traditional sense as linear polymers do;^31,33^ e.g., no “tube” can be defined around their contour, and they do not “reptate”.^33^ Rings display architecture-specific topological constraints called threadings,^34,35^ which display the puzzling property of reducing self-similarly over time.^36,37^ The development of a method to robustly and unambiguously quantify entanglements in melts of ring polymers is still an open challenge in the field of polymer physics.^38,39^

In parallel to these open questions, it is clear that the geometric design of systems with specific entanglements in their microstructure could in principle allow for the control of mesoscopic material properties.^2,40,41^ The realization of woven structures can now be achieved at both micro- and mesoscales using synthetic chemistry^42^ or 3D printing.^41^ To bypass a virtually endless trial-and-error approach, it is therefore important to be able to select the entanglement motifs to embroider in the structures in such a way that they display the desired mechanical properties.^42^ Interestingly, this problem is not too dissimilar to that of knitting socks: using solely two types of stitches (“knit” and “purl”), it is possible to create many distinct motifs and socks with distinctive elastic properties.^43,44^

Another example in which topological entanglements are abundant is in molecular biology and genome organization. Two meters of genome is packed in a 10 μm nucleus in human cells. This extreme level of packaging leads to knotting and entanglement, which are resolved by topoisomerase (Topo2), a protein that is about 50 nm in size that can identify topological knots from pure geometric entanglements in DNA molecules that are more than a thousand times bigger.^45^ By a still poorly understood “sensing” mechanism,^46,47^ Topo2 is able to reduce the topological complexity of DNA *in vivo*(46,48,49) and *in vitro*(50) without introducing more complex knots.

Inspired by Topo2’s topological sensing, which is necessarily local and unable to account for the global topology of knotted DNA, here, we investigate the possibility that there exist some geometric descriptors that correlate with the underlying topology of generic closed curves involved, for instance, in woven structures or polymer melts. To this end, we perform molecular dynamics (MD) simulations of knotted and linked semiflexible polymers in equilibrium and study the correlation between the position of the shortest knotted and linked arcs with that of four geometric descriptors: (i) regions of maximum local curvature, (ii) regions of maximum local density, (iii) regions with maximum local 1D writhe, and (iv) regions with maximum nonlocal 3D writhe. We note that while Topo2 works on a very specific polymer, the DNA double helix, here, we are interested in exploring the relationship between local geometry and global topology on generic polymers with the hope that our results may be helpful to better understand the entanglements in generic entangled polymer systems.

We discover that regions of maximum local density strongly correlate with knotted and linked arcs and outperform regions of maximum curvature. Surprisingly, we also find that this effect persists under strong confinement, where the knotted polymer is confined within a sphere smaller than its size in equilibrium. Finally, we show that 3D writhe is the best geometrical descriptors to recognize knotted arcs, and it performs consistently better than other geometric predictors. We conjecture that these local geometric descriptors could be employed to compute topological entanglements in more complex systems such as polymer melts, networks, tangles, and weavings.

## Methods

### Simulation Details

We model knotted and linked curves as semiflexible coarse-grained bead–spring polymers with *N* = 500 beads of size σ. The beads interact with each other via a purely repulsive Lennard-Jones potential
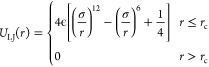
1where *r* denotes the separation between the beads and the cutoff *r*_c_ = 2^1/6^σ is chosen so that only the repulsive part of the potential is used. Nearest-neighbor monomers along the contour of the chains are connected by finitely extensible nonlinear elastic (FENE) springs as
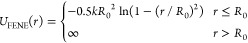
2where *k* = 30ϵ/σ^2^ is the spring constant and *R*_0_ = 1.5σ is the maximum extension of the elastic FENE bond. This choice of potentials and parameters is essential to preclude thermally driven strand crossings and therefore ensures that the global topology is preserved at all times.^51^ Finally, we add bending rigidity via a Kratky–Porod potential, *U*_bend_(θ) = *k*_θ_ (1 – cos θ), where θ is the angle formed between consecutive bonds and *k*_θ_ = 20*k*_B_*T* is the bending constant. We choose this value to mimic that of DNA, as for σ = 2.5 nm, the persistence length would be matched to *l*_p_ = 50 nm, as known for DNA.^52^ Each bead’s motion is then evolved via a Langevin equation, i.e., by adding to the Newtonian equations of motion a friction and stochastic term related by the fluctuation–dissipation relation, where the amplitude of the stochastic delta-correlated force is given by , and γ is the friction coefficient. The numerical evolution of the system is conducted using a velocity-verlet scheme with  in LAMMPS.^53^ In order to simulate knotted chains, we initialize the chain of beads using the well-known parametrization for torus knots: (*x*, *y*, *z*)(*t*) = (*R*(cos *qt* + *r*) cos *pt*, *R*(cos *qt* + *r*) sin (*pt*), – *R* sin (*qt*)) where *p* and *q* are coprime integers, *R* and *r* are two constants, and *t* ∈ (0, 2π).

In our paper, we want to compute the likelihood that some geometric features (to be defined below) yield accurate predictions of where the shortest knotted or linked segments are. To do this, we typically consider 1000 configurations taken by dumping the coordinates of the beads every 10^4^ LAMMPS steps (or 10^2^τ_LJ_). From each simulation, we obtain the fraction of instances in which our predictors (described below) correctly identify the knotted or linked arc. We then run 64 independent replicas (starting from different initial conformations) and typically plot the distribution of this fraction in the form of boxplots (see below for details).

## Results

### Geometric Descriptors

**Figure 1:**
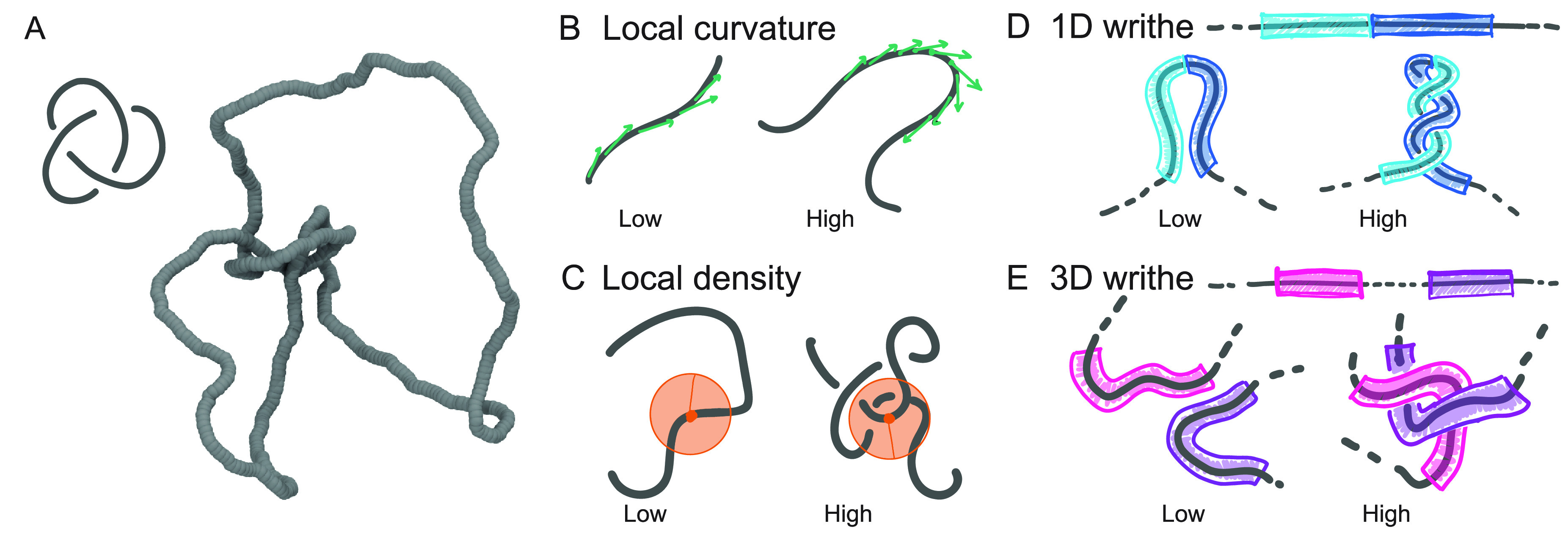
(A) Snapshot of a trefoil knot during a molecular dynamics simulation. (B–E) Illustration of the four geometric descriptors considered in this work. (B) Local curvature (eq 3), (C) local density (eq 4), (D) 1D writhe (eq 5), and (E) 3D writhe (eq 6).

As mentioned above, we consider four geometric descriptors that allow us to map polymer beads to a scalar quantity (see Figure 1 for a visual representation). They are (i) local polymer curvature (see Figure 1B)
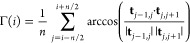
3where **t**_*j*,*j*+1_ ≡ **r**_*j*+1_ – **r**_*j*_ is the tangent vector at bead *j* and *n* = 20 an averaging window; (ii) local bead density (see Figure 1C)
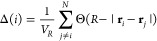
4where Θ(*x*) = 1 if *x* > 0 and 0 otherwise. In this equation, *V*_R_ = 4π*R*^3^/3 and is the volume of a sphere of radius *R*, and we take *R* = 30σ, slightly larger than a persistence length. We have checked that other sensible choices of *R* give similar results. (iii) For the local or 1D (unsigned) writhe (see Figure 1D), the equation is
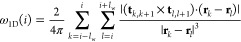
5where *l*_w_ = 50σ is the window length over which the calculation is performed. Finally, for (iv) nonlocal or 3D (unsigned) writhe (see Figure 1E), the equation is
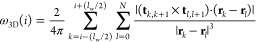
6which measures the (unsigned) entanglement of a polymer length centered at bead *i* against the rest of the polymer contour.

Equation 5 is the local generalization of the well-known “average crossing number”^54^ and has been previously used to identify supercoiled plectonemes in simulated DNA,^55,56^ branches in ring polymers,^57^ and self-entanglements in proteins.^19^Equation 6 is a generalization of eq 5 where we do not restrict the calculation of the (unsigned) writhe to occur between contiguous polymer segments. Intuitively, eqs 5 and 6 effectively compute the average number of times the contiguous (for 1D) and noncontiguous (for 3D) segments of the polymer display crossings when observed from many different directions. Accordingly, we define the beads at which our descriptors attain their maximum value as *i*_*X*_ = arg max_*i*_{*X*}, where *X* = {Δ(*i*), Γ(*i*), ω_1D_(*i*), ω_3D_(*i*)}.

**Figure 2:**
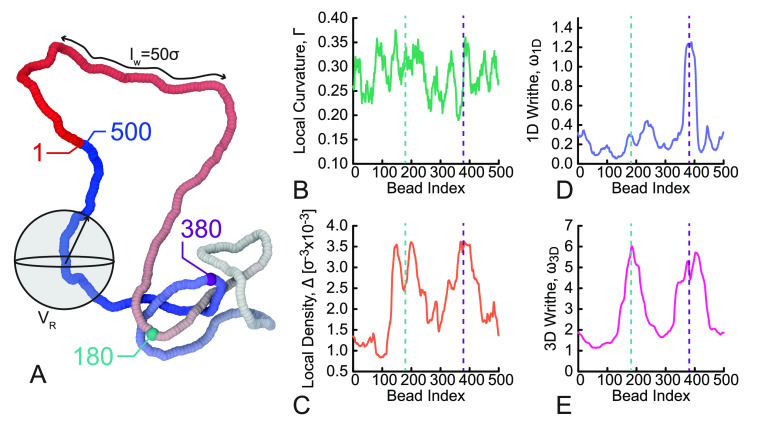
(A) Snapshot of a simulated trefoil knot conformation, color coded in terms of the bead index. The window *l*_w_ = 50σ used for the 1D and 3D writhe and the sphere volume *V*_R_ of radius *R* = 30σ used for the local density are also shown. (B–E) Curves obtained via the calculation of the geometric descriptors defined in the text and computed on the configuration in (A): (B) local curvature (eq 3), (C) local density (eq 4), (D) 1D writhe (eq 5), and (E) 3D writhe (eq 6). The beads (180 and 380) corresponding to peaks in the local density and 3D writhe are highlighted in (A–E).

Examples of typical curves that we get from the calculation of these observables on simulated polymers are shown in Figure 2. The snapshot in Figure 2A has been color coded from red to blue to identify the bead index. Beads 180 and 380 are colored green and purple to highlight the correspondence with the curves on the right of Figure 2. One can appreciate that the local curvature Γ (Figure 2B) is rather noisy and does not seem to reflect an increase in entanglements around beads 180 and 380. On the contrary, local density Δ (Figure 2C) displays three local maxima corresponding to increased density of 3D proximal segments around beads 180 and bead 380. Strikingly, 1D writhe ω_1D_ and 3D writhe ω_1D_ (Figure 2D,E) display the most intuitive and marked trends. The 1D writhe ω_1D_ is best suited to detect self-entanglements over short distances (around *l*_w_), while the 3D writhe ω_3D_ is able to detect self-entanglements over large distances. Intuitively, the peaks correspond to the location of the essential crossings of the trefoil knot.

### Knot Localization

**Figure 3:**
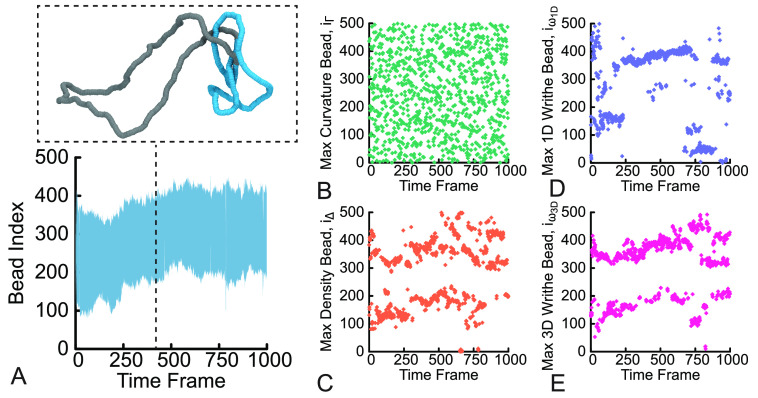
Kymographs (the evolution of geometric and topological observables over time). (A) The range of beads identified by Kymoknot that form the shortest knotted arc during one simulation of a trefoil knot is shaded in blue. The inset shows a snapshot of the simulation, corresponding to an instantaneous conformation with the shortest knotted arc color coded in blue. (B–E) The arg max values of (B) local curvature, (C) local density, (D) 1D writhe, and (E) 3D writhe at each time frame during the molecular dynamics simulation.

To identify knotted arcs in our simulated polymer we use Kymoknot,^58^ a free and open-source software to identify the topology and shortest knotted arcs of closed and open polymer chains. The algorithm works by using a minimally interfering algorithm that (in either a top-down or a bottom-up direction) truncates the polymer conformation, computes the convex hull of the remaining polymer segments, joins the termini outside the so-formed convex hull, and then calculates the Alexander determinant of the closed conformation.^3^ The result of Kymoknot is the interval within which the shortest knotted arc is located. For a polymer conformation that evolves over time, we can visualize the output of Kymoknot in a so-called kymograph. The blue shaded region in Figure 3A represents the shortest knotted arc within the simulated polymer as it fluctuates over time. For clarity, we also show a representative snapshot of the polymer at a given time frame where we have color coded the shortest knotted arc in blue. We then directly use the Kymoknot output to count how frequently the *i*_*X*_’s computed using the geometric descriptors defined above fall within the shortest knotted interval. We call this quantity the “colocalization score”, ρ_*X*_.

The key point of this work is that Kymoknot recognizes the shortest knotted arc by computing a global topological invariant (the Alexander determinant) of a suitably closed open curve. On the contrary, the quantities defined in eqs 3–6 are purely geometric and have no knowledge of the global topology of the chain. Additionally, 3 of them, Δ, Γ, and ω_1D_, are purely *local* features that can be extracted from a short polymer segment, measuring the surrounding segments in close 1D or 3D proximity.

### Localization of Knotted Arcs by Geometric Descriptors

Having described the topological and geometrical observables used in this work to identify knotted and linked arcs, we now aim to address how well the geometric descriptors can predict the location of knots along polymers. To achieve this, we first visually compare the result from Kymoknot (Figure 3A) to the ones obtained via the *i*_*X*_’s of the geometric descriptors (Figure 3B–E). We first notice that the maximum of the local curvature Γ appears to be noisy and randomly scattered along the contour. This is also the case if we do not perform the window averaging of the local curvature or if we pick beads separated by a number of beads. On the contrary, the maximum of local density, 1D writhe, and 3D writhe appear to locate near the boundaries of the shortest knotted arc identified by Kymoknot (Figure 3A). We hypothesize that this finding may be related to the concept of essential crossings^59,60^ and that our geometric predictors may thus be able to identify some of the essential crossings in the knotted chain.

**Figure 4:**
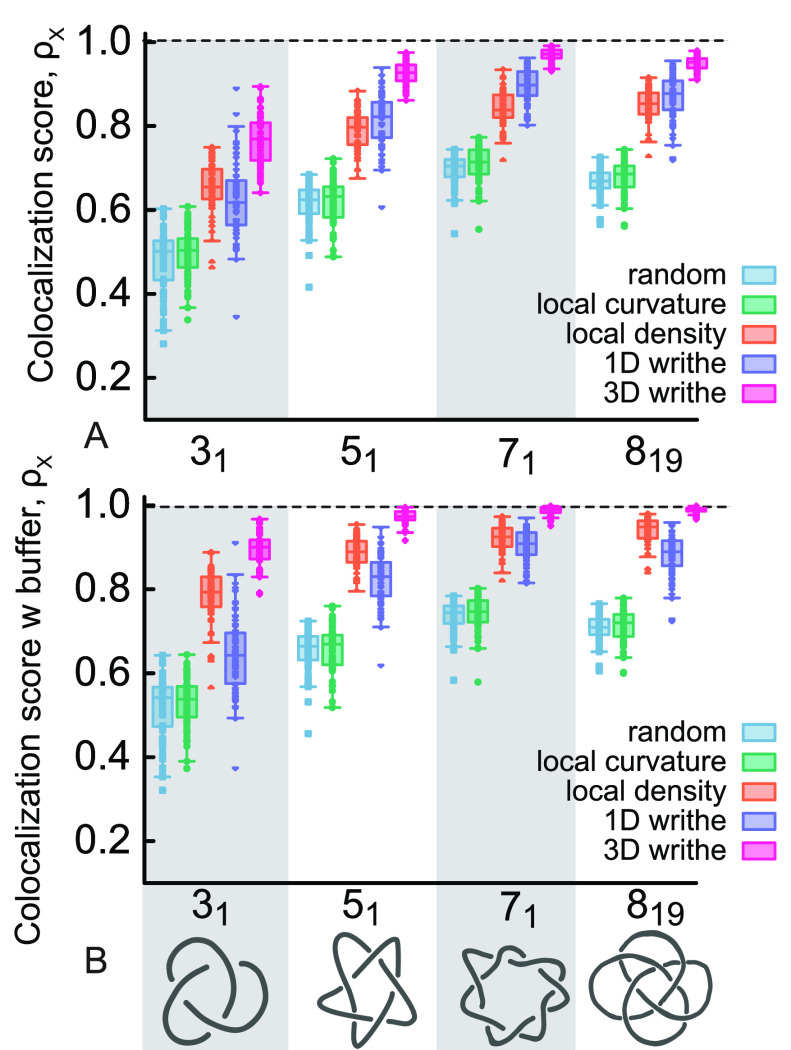
(A) Boxplots showing the colocalization score of four different knot types using the four geometric descriptors (plus a random control) over 64 replicas. Each point in the boxplot represents the colocalization score (i.e., how many times the geometric predictor is contained within the Kymoknot-detected arc) computed over 1000 conformations in each replica. (B) Same as in (A) but accounts for a “buffer” of 10 beads on either side of the boundaries detected by Kymoknot.

To more precisely quantify how well our predictors can identify the location of the shortest knotted arc, we compute the “colocalization score”, ρ_*X*_. [We recall that this was defined as the number of times that the geometrically predicted *i*_*X*_ falls within the shortest knotted interval detected by Kymoknot.] Figure 4A shows that for an unconfined, dilute polymer, ρ_Γ_ is similar to one obtained by a random choice of bead, i.e., for a trefoil ρ_rand_ ≃ 50%. Notice that a computed ρ_rand_ ≃ 0.5 means that, for our choice of parameters, the shortest knotted arc occupies about half of the polymer contour; this is due to the large polymer stiffness chosen to match that of DNA and the net effect is that the knot tends to delocalize.^3^ Interestingly, we observe a much larger colocalization score for the other geometric descriptors. More specifically, the local density descriptor *i*_Δ_ colocalizes with the knotted arc roughly ρ_Δ_ = 70% of the times for a trefoil and more than 80% for the other knot types (Figure 4A). Additionally, we find that the 3D writhe is the most accurate predictor with ρ_ω3D_ ≃ 80% for the trefoil and ρ_ω3D_ > 90% for the more complex knots.

Interestingly, if we account for a “buffer”, i.e., an additional 10 beads on either side of the knot boundaries identified by Kymoknot, we find a further increase in accuracy (see Figure 4B) with *i*_Δ_ reaching more than 80% for all knot types and 3D writhe, more than 90% for all knot types, getting close to 100% for 5_1_, 7_1_, and 8_19_. While local density improves its predictive power when including the buffer, the 1D writhe does not. Perhaps the most interesting observation from Figure 4 is that, even if more complex knots delocalize and take up a larger fraction of the polymer contour (see the random value increasing up to ≃75%), our geometric descriptors are still significantly more accurate than simply a random choice.

### Localization of Knotted Arcs under Spherical Confinement

**Figure 5:**
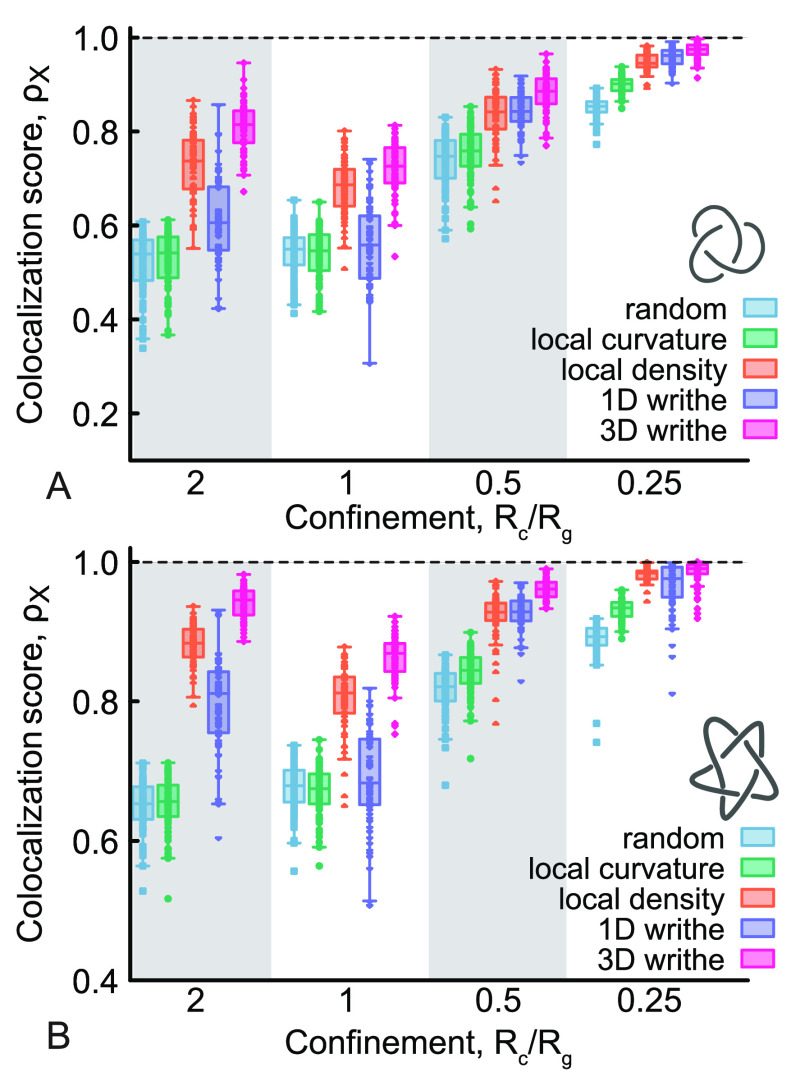
(A, B) Boxplots showing the colocalization score for a trefoil (A) and pentafoil (B) as a function of knot confinement, measured as *R*_c_/*R*_g_ where *R*_g_ is the radius of gyration of the polymer in equilibrium. As before, we compute the score over 1000 conformations and make the boxplot using one value for each of the 64 independent replicas.

Arguably, while the semiflexible nature of our chains renders knots rather delocalized over the contour, the consideration of chains that are more flexible would induce knot localization,^61,62^ which is expected to facilitate their recognition by our geometric methods. Localized knots are defined such that their subtended arc scales sublinearly with the length of the polymers, i.e., *l*_*k*_ ∼ *N*^α^ with α < 1. It was previously shown that knots in flexible chains display α ≃ 0.75.^3^ On the other hand, under spherical confinement, knots are extremely delocalized and display α ≃ 1.^3^ Thus, we ask whether our geometric predictors (and in particular the local density Δ) remain good predictors of knot location under spherical confinement. To study this regime, we enclose polymers in spherical shells with harmonic repulsive interactions with all the beads. The radius of the shell *R*_c_ is slowly reduced until the desired confinement *R*_c_/*R*_g_ (with *R*_g_ being the equilibrium radius of gyration of the polymer in dilute conditions) is attained. The polymer is then allowed to equilibrate. Finally, we measure the curves Γ(*i*), Δ(*i*), ω_1D_(*i*), and ω_3D_(*i*) as before and, in turn, the colocalization score, ρ_*X*_ (Figure 5). The only change is that we now use *R* = *R*_c_/8 to compute Δ(*i*). This is needed because under confinement the radius of gyration becomes smaller than the original value *R* = 30σ we set earlier for the dilute case. We have repeated this calculation for other sensible choices of *R* and they produce qualitatively similar results. Interestingly, we observe that Δ still outperforms a random process even at values of confinement strength *R*_c_/*R*_g_ = 0.25 for both the trefoil and pentafoil knots (see Figure 5). It is rather striking that *i*_Δ_ colocalizes with the knotted arc more than ρ_Δ_ > 95% of the time, meaning that, even under these extreme conditions of self-density, the presence of a knot can be identified via purely geometric features.

Finally, we note that the accuracy trend displays a nonmonotonic behavior as a function of confinement strength. In particular, we note a curious dip in accuracy for *R*_c_/*R*_g_ = 1. It would be interesting in the future to explore in detail the physical origin of this behavior.

### Link Localization by Geometric Descriptors

In the last part of this paper, we consider links as prototypical examples of generic entangled chains. We perform MD simulations of two *N* = 500 bead-spring Kremer–Grest polymer chains tied in a simple Hopf link. We then measure the shortest linked portion using the method described in refs (63−66) and compare the resulting segment with the ones given by our geometric descriptors. Briefly, the algorithm works as follows: from a pair of linked curves with topology τ computed using the two-variable Alexander polynomial,^63^ it is possible to obtain the shortest physical link by looking at all possible pairs of subchains (γ_1_, γ_2_) on the condition that they display the same topology as the original link. The algorithm employs a top-down search scheme on the basis of a bisection method and outputs the index of the beads in chain 1 and chain 2. We then count how likely it is that the *i*_*X*_’s obtained using the geometric predictors fall within the shortest linked regions of the two chains.

**Figure 6:**
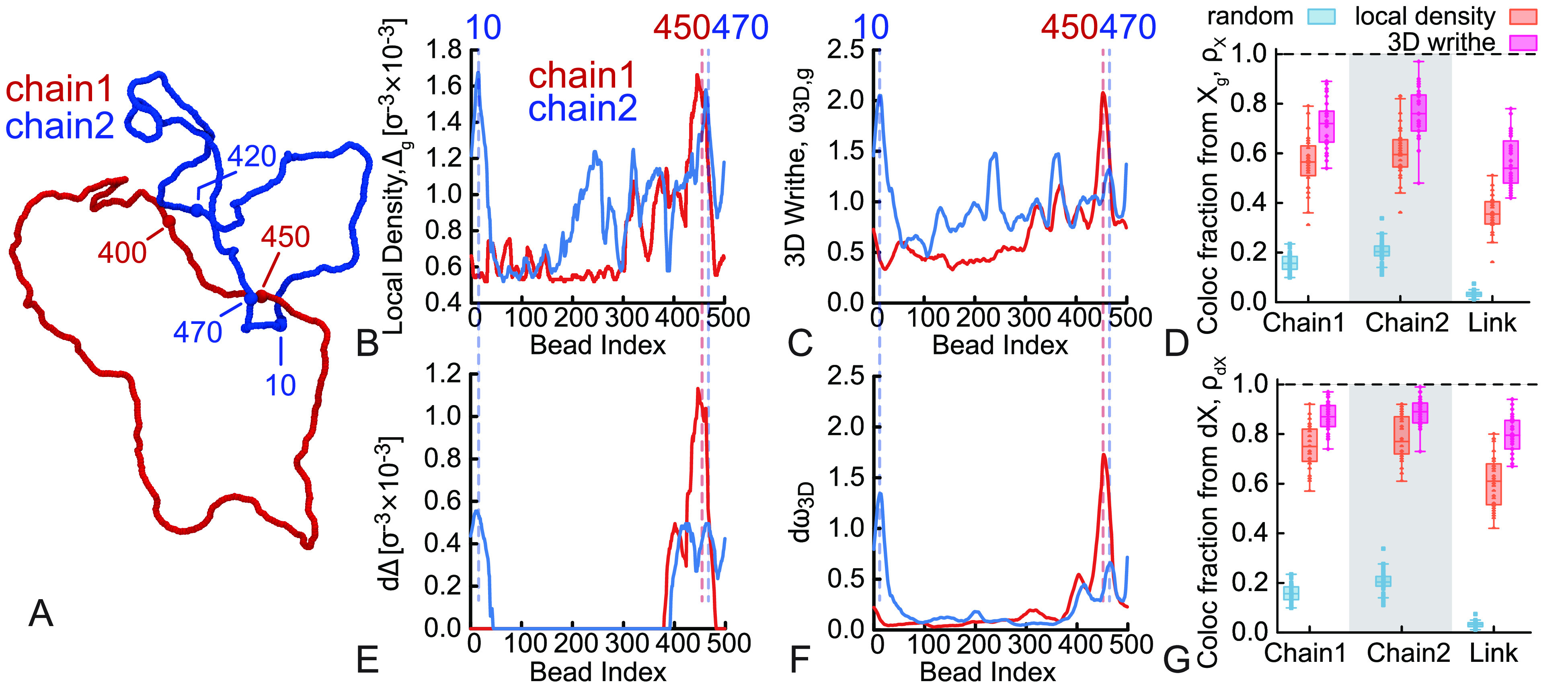
(A) Snapshot of a MD simulation of two rings, each *N* = 500 beads long, tied in a Hopf link. Some beads are highlighted and made larger for visualization purposes. The algorithm introduced in ref (63) detected the shortest linked segments spanning beads 421 to 460 for chain 1 and 461 to 20 for chain 2 (across the periodic boundary). (B) Local density, Δ_g_, and (C) 3D writhe, ω_3D,g_, computed considering all the beads in the system. (D) Colocalization score for the single chain components and the overall link from the “global” predictors, *X*_g_. (E) Local density, *d*Δ, and (F) 3D writhe, *d* ω_3D_, computed from the difference of global and self-components of the predictors: d*X*(*i*) = *X*_g_(*i*) – *X*_s_(*i*). (G) Colocalization score for the single chain components and the overall link from the differential predictors, d*X*.

We here compare the results from the link localization algorithm with our two best performing descriptors, i.e., the local density, Δ, and the 3D writhe, ω_3D_. Since we now consider two chains, we can define Δ(*i*) and ω_3D_(*i*) as “self” (when computing them considering only the chain that hosts the *i*^th^ segment) or as “global” (when considering all beads in the system in the calculation). The trend of *X*_s_(*i*) reflects the entanglements of the chain with itself while *X*_g_(*i*) mirrors any entanglement segment to which *i* is subjected. In Figure 6A–C, we show that, for a randomly chosen simulation snapshot, the global features *X*_g_(*i*) display several maxima and the higher ones correspond to the beads forming the link. For the particular snapshot in Figure 6A, the link localization algorithm^63^ detects the shortest linked arc in chain 1 (red in the figure) to be 421–460 and the shortest linked arc in chain 2 (blue in the figure) to be 461–20 (through periodic boundary conditions at *N* = 500). We highlighted the positions of these beads in Figure 6A–C,E,F, to show the agreement with Δ_g_ and ω_3D,g_.

The colocalization score calculated on the global geometric predictors (shown in Figure 6D) suggests that these features correlate well with the location of the link. As expected, we do not see any significant difference when comparing the accuracy of chain 1 and chain 2, and we observe that the colocalization score for the total link, i.e., the conditional probability that both linked segments contain *i*_*X*_g__, appears to be roughly the product of the two colocalization scores for the single components. Importantly, Figure 6D shows that the geometric predictors significantly outperform the random prediction (even by a factor of 5 or more).

We then noted that the difference of the global and the self-components of the geometric predictors, defined as d*X*(*i*) = *X*_g_(*i*) – *X*_s_(*i*), significantly decrease the fluctuations of the curves. Intuitively, d*X*(*i*) counts the contributions of interchain segments on the segment *i* (see Figure 6E,F). Strikingly, we find that *i*_d*X*_, i.e., the bead hosting the maximum value of the difference d*X*, yields an even better colocalization score with values around 90% for the individual link components and 80% for the total link (Figure 6G). The ratio of the localization accuracy of the geometric predictors and the random choice is now 10 or more. Arguably, this means that the interchain correlations are the most important contribution to the entanglements. This is also in line with the situation in entangled polymer melts, where total density fluctuations are typically small, while interchain density fluctuations are more informative of the system dynamics.^67,68^

## Discussion and Conclusions

What makes a curve knotted? Inside our cells, how do certain proteins recognize complex topologies by scanning the DNA locally? How can we unambiguously identify relevant entanglements in polymeric systems? In this work, we started from the hypothesis that knotted and linked curves in 3D may harbor some geometric features that correlate with the underlying topology. To this end, we have performed MD simulations of knotted and linked curves and have analyzed four geometric predictors: (i) local curvature, (ii) local density, (iii) 1D writhe, and (iv) 3D writhe. We used the geometric predictors to locate the shortest knotted and linked arcs and compared these predictions to the ones given by state-of-the-art knot and link localization algorithms (refs (3, 58, and 63)).

We discovered that local curvature is equivalent to randomly choosing a bead within the contour. This is interesting as there are models arguing that Topoisomerase, a protein involved in simplifying knots in DNA, may sense curvature to locate a knotted segment.^69^ Our work suggests that this would be a poor search strategy and would yield a rather inefficient topological simplification pathway. Admittedly, our model does not capture the torsional rigidity and the double-helical structure of DNA and we thus refrain from arguing that our results clarify the search strategy of Topoisomerases on DNA. At the same time, our results suggest that, in polymer melts and other generic thermally driven entangled systems, such as weavings, the points of maximum curvature of the filaments are not necessarily the most entangled.

On the other hand, we find that local density is a far better geometric predictor of topologically complex states. In our simulations, the bead in the polymer with the largest number of neighbors (largest local density) is often also part of the knotted or linked segment (with an accuracy of ≃80% for simple knots and the Hopf link and up to 90% for more complex knots or under confinement). This is rather striking in that the calculation of local density is restricted to beads that are 3D proximal to bead *i* and there is no information on the global topology of the curve. One consequence of our findings is that sensing the local density of DNA segments could be a good strategy for Topoisomerase to quickly locate knotted and entangled arcs. Such a binding strategy may be naturally realized by a protein design that presents abundant positively charged amino acids on the surface of the protein in such a way as to maximize unspecific interactions with negatively charged DNA. Indeed, Topoisomerases typically present a positively charged area in the region of DNA binding that is far larger than the one needed to bind DNA.^70,71^ Again, we stress that our polymer model does not fully capture DNA’s complexity. In the future, we aim to perform a similar analysis on models that can capture twist^72,73^ to quantify the impact of torsional rigidity on these metrics. Furthermore, it has been suggested that in knotted and closed DNA there may be an interplay of both knots and plectonemes; in this case, the geometric descriptors measured here may struggle to identify the essential crossings of the knot from the writhe of the plectoneme. Future studies will illuminate this issue. In spite of the limitations of our present model in modeling DNA, we conjecture that our results may be used to quantify entanglement motifs in tangled and weaved structures.^41,42^ For instance, we expect that the pattern of local density along the entangled curves will be motif-dependent and that there may be a relationship between these patterns and the corresponding mesoscopic elasticity. Again, we hope that future work will explore this direction further.

Finally, we discover that 3D writhe is our best descriptor with a consistently high (≳90%) accuracy in identifying the knotted and linked arcs. This observation is less striking than the one for the local density as 3D writhe is not (strictly speaking) a local geometric predictor. In other words, the calculation of 3D writhe has to scale as *N*^2^ while the local curvature, density, and 1D writhe scale as *N*. We note that local density can make use of neighbor lists; hence, why we claim it could scale faster than *N*^2^.

In line with this, we note that state-of-the-art algorithms that search for knotted and linked segments on polymeric systems^58,63,74^ or proteins^20,75,76^ require a considerable amount of computational time. For instance, when run on a single CPU, knot localization on our *N* = 500 chain in dilute conditions takes about 2 ms but under confinement takes up to 300 ms per conformation. On the other hand, the calculation of the local density profile takes on average 0.3 ms. Similarly, link localization for our two *N* = 500 chains takes up to a minute even in dilute conditions on a single conformation. On the contrary, the calculation of the local density profile for the same link takes 30 ms per conformation. For this reason, we argue that adding a preliminary search step using geometric predictors, before launching a full blown topological search scheme, could be a way to render search algorithms more efficient in the future.

It is appropriate here to highlight that entanglements are among the most elusive and slippery topics in polymer science. Algorithms such as an isoconfigurational mean path^29^ and primitive path analysis^28^ are the “gold standard” to quantify relevant entanglements in polymeric systems and yet they fail in the case of ring polymers.^31^ We hope that the geometric descriptors proposed here may be a complement to these tools and could be used to identify entanglements in complex polymeric systems. We speculate that (interchain) local density and 1D and 3D writhe as defined in this work may yield interesting results not only in melts of ring polymers but also in molecular (and periodic) weavings.^2,40−42^ We expect that different entanglement motifs are associated with distinct patterns of our geometric observables. In turn, they may be used to predict the global elastic response of the entangled network to certain perturbations. To the best of our knowledge, these metrics have not yet been tried on polymer melts or molecular weavings.

One intriguing application of our results is on Olympic gels.^16,77−79^ Indeed, there is no simple way to compute the extension of three or more components of the Gauss linking number, known as the Milnor’s triple linking number,^80^ on the systems of ring polymers. This means that it is extremely challenging to unambiguously discern three physically inseparable Borromean rings from three unlinked and physically separable rings. Systems made of interlinked “Olympic” rings,^77^ such as the naturally occurring Kinetoplast DNA^16,81^ or synthetic equivalents,^79^ are likely to display Borromean and higher order Brunnian configurations of interlinked rings.^82^ This means that computing the pairwise (Gauss) linking number between rings is likely not enough to predict the mesoscopic elasticity of Olympic gels, as this metric completely neglects contributions from Brunnian links. We hope that our geometric predictors may be able to offer an alternative to the lack of (simple) topological invariants to characterize these elusive conformations. For instance, a step toward this goal in the near future would be to study the behavior of our geometric predictors in simple Borromean rings in dilute conditions.

Finally, we note that the data generated by our geometric predictors lend themselves fittingly to be used as input features for machine learning algorithms, e.g., neural networks, to identify knots and entanglements. This is because our predictors are invariant under translations and rotations of the conformation and under relabeling of the beads. In the future, we thus aim to couple our geometric observables to Machine Learning, as recently done in ref (83), to identify and localize knots and entanglements in more complex systems.

## References

[ref1] Panagiotou E.; Kröger M.; Millett K. C. Writhe and mutual entanglement combine to give the entanglement length. Physical Review E - Statistical, Nonlinear, and Soft Matter Physics 2013, 88, 32–35. 10.1103/PhysRevE.88.062604. 24483478

[ref2] Igram S.; Millett K. C.; Panagiotou E. Resolving critical degrees of entanglement in Olympic ring systems. Journal of Knot Theory and its Ramifications 2016, 25, 1650081 10.1142/S0218216516500814.

[ref3] Tubiana L.; Orlandini E.; Micheletti C. Multiscale entanglement in ring polymers under spherical confinement. Phys. Rev. Lett. 2011, 107, 188302 10.1103/PhysRevLett.107.188302. 22107680

[ref4] Goundaroulis D.; Dorier J.; Benedetti F.; Stasiak A. Studies of global and local entanglements of individual protein chains using the concept of knotoids. Sci. Rep. 2017, 7, 6309 10.1038/s41598-017-06649-3. 28740166 PMC5524787

[ref6] Goundaroulis D.; Lieberman Aiden E.; Stasiak A. Chromatin Is Frequently Unknotted at the Megabase Scale. Biophys. J. 2020, 118, 2268–2279. 10.1016/j.bpj.2019.11.002. 31818464 PMC7202934

[ref7] Panagiotou E.; Kauffman L. H. Knot polynomials of open and closed curves. Proceedings of the Royal Society A: Mathematical, Physical and Engineering Sciences 2020, 476, 20200124 10.1098/rspa.2020.0124. PMC7482204 32922152

[ref8] Machon T.; Alexander G. P. Knots and nonorientable surfaces in chiral nematics. Proc. Natl. Acad. Sci. U.S.A. 2013, 110, 14174–14179. 10.1073/pnas.1308225110. 23940365 PMC3761586

[ref9] O’Holleran K.; Dennis M. R.; Flossmann F.; Padgett M. J. Fractality of light’s darkness. Phys. Rev. Lett. 2008, 100, 053902 10.1103/PhysRevLett.100.053902. 18352372

[ref10] Dennis M.; King R.; Jack B.; O’Holleran K.; Padgett M. Isolated optical vortex knots. Nat. Phys. 2010, 6, 118–121. 10.1038/nphys1504.

[ref11] Laing C. E.; Ricca R. L.; Sumners D. W. L. Conservation of writhe helicity under anti-parallel reconnection. Sci. Rep. 2015, 5, 9224 10.1038/srep09224. 25820408 PMC4377626

[ref12] Smrek J.; Grosberg A. Y. A novel family of space-filling curves in their relation to chromosome conformation in eukaryotes. Physica A 2013, 392, 6375–6388. 10.1016/j.physa.2013.08.014.

[ref13] Siebert J. T.; Kivel A. N.; Atkinson L. P.; Stevens T. J.; Laue E. D.; Virnau P. Are there knots in chromosomes?. Polymers 2017, 9, 317 10.3390/polym9080317. PMC6418659 30971010

[ref14] Michieletto D.; Orlandini E.; Marenduzzo D. Epigenetic Transitions and Knotted Solitons in Stretched Chromatin. Sci. Rep. 2017, 7, 14642 10.1038/s41598-017-13916-w. 29116102 PMC5676697

[ref15] Marenduzzo D.; Orlandini E.; Stasiak A.; Sumners D.; Tubiana L.; Micheletti C. DNA-DNA interactions in bacteriophage capsids are responsible for the observed DNA knotting. Proc. Natl. Acad. Sci. U.S.A. 2009, 106, 22269–74. 10.1073/pnas.0907524106. 20018693 PMC2799769

[ref16] Klotz A. R.; Soh B. W.; Doyle P. S. Equilibrium structure and deformation response of 2D kinetoplast sheets. Proc. Natl. Acad. Sci. U.S.A. 2020, 117, 121–127. 10.1073/pnas.1911088116. 31811027 PMC6955370

[ref17] Klotz A. R.; Soh B. W.; Doyle P. S. An experimental investigation of attraction between knots in a stretched DNA molecule. EPL 2020, 129, 68001 10.1209/0295-5075/129/68001.

[ref18] Polson J. M.; Garcia E. J.; Klotz A. R. Flatness and intrinsic curvature of linked-ring membranes. Soft Matter 2021, 17, 10505–10515. 10.1039/D1SM01307F. 34755161

[ref19] Baiesi M.; Orlandini E.; Seno F.; Trovato A. Exploring the correlation between the folding rates of proteins and the entanglement of their native states. Journal of Physics A: Mathematical and Theoretical 2017, 50, 504001 10.1088/1751-8121/aa97e7.

[ref20] Dabrowski-Tumanski P.; Sulkowska J. I. Topological knots and links in proteins. Proc. Natl. Acad. Sci. U. S. A. 2017, 114, 3415–3420. 10.1073/pnas.1615862114. 28280100 PMC5380043

[ref21] Marenduzzo D.; Micheletti C.; Orlandini E. Biopolymer organization upon confinement. J. Phys.: Condens. Matter 2010, 22, 283102 10.1088/0953-8984/22/28/283102. 21399272

[ref22] Wu Q.; Rauscher P. M.; Lang X.; Wojtecki R. J.; De Pablo J. J.; Hore M. J.; Rowan S. J. Poly[n]catenanes: Synthesis of molecular interlocked chains. Science 2017, 358, 1434–1439. 10.1126/science.aap7675. 29192134

[ref23] Rauscher P. M.; Rowan S. J.; De Pablo J. J. Topological Effects in Isolated Poly[n]catenanes: Molecular Dynamics Simulations and Rouse Mode Analysis. ACS Macro Lett. 2018, 7, 938–943. 10.1021/acsmacrolett.8b00393. 35650969

[ref24] Goldstein R. E.; Moffatt H. K.; Pesci A. I.; Ricca R. L. Soap-film Mobius strip changes topology with a twist singularity. Proc. Natl. Acad. Sci. U.S.A. 2010, 107, 21979–21984. 10.1073/pnas.1015997107.

[ref25] Machon T.; Alexander G. P.; Goldstein R. E.; Pesci A. I. Instabilities and Solitons in Minimal Strips. Phys. Rev. Lett. 2016, 117, 017801 10.1103/PhysRevLett.117.017801. 27419593

[ref26] Kamien R. D. The geometry of soft materials: a primer. Rev. Mod. Phys. 2002, 74, 953–971. 10.1103/RevModPhys.74.953.

[ref27] Dennis M. R.; Hannay J. H. Geometry of Calugareanu’s theorem. Proc. R. Soc. A 2005, 461, 3245–3254. 10.1098/rspa.2005.1527.

[ref28] Everaers R. Rheology and Microscopic Topology of Entangled Polymeric Liquids. Science 2004, 303, 823–826. 10.1126/science.1091215. 14764875

[ref29] Bisbee W.; Qin J.; Milner S. T. Finding the tube with isoconfigurational averaging. Macromolecules 2011, 44, 8972–8980. 10.1021/ma2012333.

[ref30] McLeish T. Polymers without beginning or end. Science 2002, 297, 2005–6. 10.1126/science.1076810. 12242428

[ref31] Halverson J. D.; Lee W. B.; Grest G. S.; Grosberg A. Y.; Kremer K. Molecular dynamics simulation study of nonconcatenated ring polymers in a melt. I. Statics. J. Chem. Phys. 2011, 134, 204904 10.1063/1.3587137. 21639474

[ref32] Rosa A.; Everaers R. Ring polymers in the melt state: the physics of crumpling. Phys. Rev. Lett. 2014, 112, 118302 10.1103/PhysRevLett.112.118302. 24702424

[ref33] Halverson J. D.; Lee W. B.; Grest G. S.; Grosberg A. Y.; Kremer K. Molecular dynamics simulation study of nonconcatenated ring polymers in a melt. II. Dynamics. J. Chem. Phys. 2011, 134, 204905 10.1063/1.3587138. 21639475

[ref34] Michieletto D.; Marenduzzo D.; Orlandini E.; Alexander G. P.; Turner M. S. Threading Dynamics of Ring Polymers in a Gel. ACS Macro Lett. 2014, 3, 255–259. 10.1021/mz500060c. 35590516

[ref35] Gómez L. R.; García N. A.; Pöschel T. Packing structure of semiflexible rings. Proc. Natl. Acad. Sci. U.S.A. 2020, 117, 3382–3387. 10.1073/pnas.1914268117. 32024763 PMC7035624

[ref36] Ge T.; Panyukov S.; Rubinstein M. Self-Similar Conformations and Dynamics in Entangled Melts and Solutions of Nonconcatenated Ring Polymers. Macromolecules 2016, 49, 708–722. 10.1021/acs.macromol.5b02319. 27057066 PMC4819263

[ref37] Michieletto D.; Sakaue T. Dynamical Entanglement and Cooperative Dynamics in Entangled Solutions of Ring and Linear Polymers. ACS Macro Lett. 2021, 10, 129–134. 10.1021/acsmacrolett.0c00551. 35548984

[ref38] Smrek J.; Grosberg A. Y. Minimal Surfaces on Unconcatenated Polymer Rings in Melt. ACS Macro Lett. 2016, 5, 750–754. 10.1021/acsmacrolett.6b00289. 35614671

[ref39] Landuzzi F.; Nakamura T.; Michieletto D.; Sakaue T. Persistence homology of entangled rings. Physical Review Research 2020, 2, 33529 10.1103/PhysRevResearch.2.033529.

[ref40] Evans M. E.; Roth R. Shaping the skin: The interplay of mesoscale geometry and corneocyte swelling. Phys. Rev. Lett. 2014, 112, 038102 10.1103/PhysRevLett.112.038102. 24484167

[ref41] Oster M.; Dias M. A.; de Wolff T.; Evans M. E. Reentrant tensegrity: A three-periodic, chiral, tensegrity structure that is auxetic. Science Advances 2021, 7, 1–7. 10.1126/sciadv.abj6737. PMC8664249 34890240

[ref42] August D. P.; Dryfe R. A.; Haigh S. J.; Kent P. R.; Leigh D. A.; Lemonnier J. F.; Li Z.; Muryn C. A.; Palmer L. I.; Song Y.; Whitehead G. F.; Young R. J. Self-assembly of a layered two-dimensional molecularly woven fabric. Nature 2020, 588, 429–435. 10.1038/s41586-020-3019-9. 33328664

[ref43] Matsumoto E. A.; Liang H.; Mahadevan L. Topology, Geometry, and Mechanics of Z -Plasty. Phys. Rev. Lett. 2018, 120, 068101 10.1103/PhysRevLett.120.068101. 29481240

[ref44] Markande S. G.; Matsumoto E. A. Knotty knits are tangles on tori. arXiv, February 4, 2020, arxiv:2002.01497. http://arxiv.org/abs/2002.01497 (accessed 2022-06-10).

[ref45] Wang J. C. DNA topoisomerases. Annu. Rev. Biochem. 1985, 54, 665–97. 10.1146/annurev.bi.54.070185.003313. 2992360

[ref46] Martínez-García B.; Fernández X.; Díaz-Ingelmo O.; Rodríguez-Campos A.; Manichanh C.; Roca J. Topoisomerase II minimizes DNA entanglements by proofreading DNA topology after DNA strand passage. Nucleic Acids Res. 2014, 42, 1821–1830. 10.1093/nar/gkt1037. 24185700 PMC3919613

[ref47] Michieletto D.; Fosado Y. A. G.; Melas E.; Baiesi M.; Tubiana L.; Orlandini E. Dynamic and facilitated binding of topoisomerase accelerates topological relaxation. Nucleic Acids Res. 2022, 50, 4659–4668. 10.1093/nar/gkac260. PMC9071436 35474478

[ref48] Piskadlo E.; Oliveira R. A. A topology-centric view on mitotic chromosome architecture. International Journal of Molecular Sciences 2017, 18, 2751 10.3390/ijms18122751. PMC5751350 29258269

[ref49] Valdés A.; Segura J.; Dyson S.; Martínez-García B.; Roca J. DNA knots occur in intracellular chromatin. Nucleic Acids Res. 2018, 46, 650–660. 10.1093/nar/gkx1137. 29149297 PMC5778459

[ref50] Rybenkov V. V.; Ullsperger C.; Vologodskii A. V.; Cozzarelli N. R. Simplification of DNA Topology Below Equilibrium Values by by Type II Topoisomerases. Science 1997, 277, 690–693. 10.1126/science.277.5326.690. 9235892

[ref51] Kremer K.; Grest G. S. Dynamics of entangled linear polymer melts: A molecular-dynamics simulation. J. Chem. Phys. 1990, 92, 5057–5086. 10.1063/1.458541.

[ref52] Calladine C. R.; Drew H.; Luisi F. B.; Travers A. A.; Bash E. Understanding DNA: the molecule and how it works; Elsevier Academic Press, 1997; Vol. 1.

[ref53] Plimpton S. Fast Parallel Algorithms for Short-Range Molecular Dynamics. J. Comp. Phys. 1995, 117, 1–19. 10.1006/jcph.1995.1039.

[ref54] Stasiak A.; Katritch V.; Bednar J.; Michoud D.; Dubochet J. Electrophoretic mobility of DNA knots. Nature 1996, 384, 122 10.1038/384122a0. 8906784

[ref55] Klenin K.; Langowski J. Computation of writhe in modeling of supercoiled DNA. Biopolymers 2000, 54, 307–17. 10.1002/1097-0282(20001015)54:5<307::AID-BIP20>3.0.CO;2-Y. 10935971

[ref56] Smrek J.; Garamella J.; Robertson-Anderson R.; Michieletto D. Topological tuning of DNA mobility in entangled solutions of supercoiled plasmids. Science Advances 2021, 7, 1–28. 10.1126/sciadv.abf9260. PMC8115916 33980492

[ref57] Michieletto D. On the tree-like structure of rings in dense solutions. Soft Matter 2016, 12, 9485–9500. 10.1039/C6SM02168A. 27781227

[ref58] Tubiana L.; Polles G.; Orlandini E.; Micheletti C. KymoKnot: A web server and software package to identify and locate knots in trajectories of linear or circular polymers. Eur. Phys. J. E 2018, 41, 72 10.1140/epje/i2018-11681-0. 29884956

[ref59] Suma A.; Micheletti C. Pore translocation of knotted DNA rings. Proc. Natl. Acad. Sci. U.S.A. 2017, 114, E2991–E2997. 10.1073/pnas.1701321114. 28351979 PMC5393256

[ref60] Coronel L.; Suma A.; Micheletti C. Dynamics of supercoiled DNA with complex knots: Large-scale rearrangements and persistent multi-strand interlocking. Nucleic Acids Res. 2018, 46, 7533–7541. 10.1093/nar/gky523. 29931074 PMC6125635

[ref61] Grosberg A. Y.; Rabin Y. Metastable tight knots in a wormlike polymer. Phys. Rev. Lett. 2007, 99, 217801 10.1103/PhysRevLett.99.217801. 18233259

[ref62] Tubiana L.; Rosa A.; Fragiacomo F.; Micheletti C. Spontaneous Knotting and Unknotting of Flexible Linear Polymers: Equilibrium and Kinetic Aspects. Macromolecules 2013, 46, 3669–3678. 10.1021/ma4002963.

[ref63] Caraglio M.; Micheletti C.; Orlandini E. Physical Links: Defining and detecting inter-chain entanglement. Sci. Rep. 2017, 7, 1156 10.1038/s41598-017-01200-w. 28442725 PMC5430864

[ref64] Caraglio M.; Micheletti C.; Orlandini E. Mechanical pulling of linked ring polymers: Elastic response and link localisation. Polymers 2017, 9, 327 10.3390/polym9080327. PMC6418824 30971003

[ref65] Caraglio M.; Orlandini E.; Whittington S. G. Translocation of links through a pore: Effects of link complexity and size. Journal of Statistical Mechanics: Theory and Experiment 2020, 2020, 043203 10.1088/1742-5468/ab7a20.

[ref66] Amici G.; Caraglio M.; Orlandini E.; Micheletti C. Topologically Linked Chains in Confinement. ACS Macro Lett. 2019, 8, 442–446. 10.1021/acsmacrolett.9b00114. 35651129

[ref67] Tsang B.; Dell Z. E.; Jiang L.; Schweizer K. S.; Granick S. Dynamic cross-correlations between entangled biofilaments as they diffuse. Proc. Natl. Acad. Sci. U.S.A. 2017, 114, 3322–3327. 10.1073/pnas.1620935114. 28283664 PMC5380065

[ref68] Dell Z. E.; Schweizer K. S. Intermolecular structural correlations in model globular and unconcatenated ring polymer liquids. Soft Matter 2018, 14, 9132–9142. 10.1039/C8SM01722K. 30407479

[ref69] Burnier Y.; Weber C.; Flammini A.; Stasiak A. Local selection rules that can determine specific pathways of DNA unknotting by type II DNA topoisomerases. Nucleic Acids Res. 2007, 35, 5223–5231. 10.1093/nar/gkm532. 17670794 PMC1976442

[ref70] Dong K. C.; Berger J. M. Structural basis for gate-DNA recognition and bending by type IIA topoisomerases. Nature 2007, 450, 1201–1205. 10.1038/nature06396. 18097402

[ref71] Cabral J. H. M.; Jackson A. P.; Smith C. V.; Shikotra N.; Maxwell A.; Liddington R. C. Crystal structure of the breakage-reunion domain of DNA gyrase. Nature 1997, 388, 903–906. 10.1038/42294. 9278055

[ref72] Ouldridge T. E.; Hoare R. L.; Louis A. a.; Doye J. P. K.; Bath J.; Turberfield A. J. Optimizing DNA nanotechnology through coarse-grained modeling: A two-footed DNA walker. ACS Nano 2013, 7, 2479–2490. 10.1021/nn3058483. 23414564

[ref73] Brackley C. A.; Morozov A. N.; Marenduzzo D. Models for twistable elastic polymers in Brownian dynamics, and their implementation for LAMMPS. J. Chem. Phys. 2014, 140, 135103 10.1063/1.4870088. 24712817

[ref74] Tubiana L.; Orlandini E.; Micheletti C. Probing the Entanglement and Locating Knots in Ring Polymers: A Comparative Study of Different Arc Closure Schemes. Prog. Theor. Phys. Suppl. 2011, 191, 192–204. 10.1143/PTPS.191.192.

[ref75] Dabrowski-Tumanski P.; Niemyska W.; Pasznik P.; Sulkowska J. I. LassoProt: server to analyze biopolymers with lassos. Nucleic acids research 2016, 44, W383–W389. 10.1093/nar/gkw308. 27131383 PMC4987892

[ref76] Dabrowski-Tumanski P.; Rubach P.; Niemyska W.; Gren B. A.; Sulkowska J. I. Topoly: Python package to analyze topology of polymers. Briefings in Bioinformatics 2021, 22, bbaa196 10.1093/bib/bbaa196. 32935829 PMC8138882

[ref77] de Gennes P. G. Scaling concepts in polymer physics; Cornell University Press, 1979.

[ref78] Kim Y. S.; Kundukad B.; Allahverdi A.; Nordensköld L.; Doyle P. S.; Van Der Maarel J. R. Gelation of the genome by topoisomerase II targeting anticancer agents. Soft Matter 2013, 9, 1656–1663. 10.1039/C2SM27229F.

[ref79] Krajina B. A.; Zhu A.; Heilshorn S. C.; Spakowitz A. J. Active DNA Olympic Hydrogels Driven by Topoisomerase Activity. Phys. Rev. Lett. 2018, 121, 148001 10.1103/PhysRevLett.121.148001. 30339454

[ref80] Polyak M. On Milnor ’s triple linking number. C. R. Acad. Sc. Paris 1997, 325, 77–82. 10.1016/S0764-4442(97)83937-7.

[ref81] Chen J.; Rauch C. A.; White J. H.; Englund P. T.; Cozzarelli N. The topology of the kinetoplast DNA network. Cell 1995, 80, 61–9. 10.1016/0092-8674(95)90451-4. 7813018

[ref82] Michieletto D.; Marenduzzo D.; Orlandini E. Is the Kinetoplast DNA a Percolating Network of Linked Rings at its Critical Point?. Physical Biology 2015, 12, 036001 10.1088/1478-3975/12/3/036001. 25970016

[ref83] Vandans O.; Yang K.; Wu Z.; Dai L. Identifying knot types of polymer conformations by machine learning. Phys. Rev. E 2020, 101, 022502 10.1103/PhysRevE.101.022502. 32168694

